# Good for the Self: Self-Compassion and Other Self-Related Constructs in Relation to Symptoms of Anxiety and Depression in Non-clinical Youths

**DOI:** 10.1007/s10826-015-0235-2

**Published:** 2015-06-19

**Authors:** Peter Muris, Cor Meesters, Anna Pierik, Bo de Kock

**Affiliations:** Clinical Psychological Science, Faculty of Psychology and Neuroscience, Maastricht University, P.O. Box 616, 6200 MD Maastricht, The Netherlands; Stellenbosch University, Stellenbosch, South Africa

**Keywords:** Self-compassion, Self-efficacy, Self-esteem, Anxiety and depression, Adolescents

## Abstract

This study examined relationships among self-compassion, self-esteem, and self-efficacy and symptoms of anxiety disorders and depression in a sample of 132 non-clinical adolescents aged 12–17 years. The results first of all indicated that the Shortened Self-Compassion Scale for Adolescents was reliable (i.e., all Cronbach’s alphas were >.70) and valid in terms of both construct (as demonstrated by a principal components analysis which revealed the hypothesized three-factor structure) and concurrent validity (i.e., as shown by means of positive correlations with self-esteem and self-efficacy). Further, the expected negative correlations were found between self-compassion and anxiety and depression, indicating that higher levels of this self-related construct are associated with lower symptom levels, and vice versa. Of the three components of self-compassion, mindfulness appeared most convincingly related to symptoms of anxiety and depression. Finally, when controlling for other self-related constructs, self-compassion no longer accounted for a significant proportion in the variance of symptom levels. In contrast, self-esteem (depression) and in particular self-efficacy (anxiety and depression) did show unique explanatory power.

## Introduction

Self-compassion is a relatively new self-related concept that involves “being open to and moved by one’s own suffering, experiencing feelings of caring and kindness toward oneself, taking an understanding and non-judgmental attitude toward one’s inadequacies and failures, and recognizing that one’s own experience is part of the common human experience” (Neff 2003b; p. 224). Further exploration has revealed that self-compassion essentially consists of six basic elements. Three elements are positive indicators of self-compassion, namely (1) self-kindness, which refers to the tendency to be caring and understanding with oneself when confronted with personal failures, problems, and stress; (2) common humanity, which is concerned with the inclination to recognize that one’s failure, problems, and stress are a normal part of human life; and (3) mindfulness, which has to do with the ability of not becoming too absorbed by one’s difficulties and associated negative feelings so that it is possible to retain a healthy balance between what goes right and what goes wrong. The other three elements are negative indicators of the construct and in essence the counterparts of the first three components, which are labelled as (4) self-judgment, (5) isolation, and (6) over-identification (Neff 2003a). From a psychological perspective, self-compassion is considered to be a construct of interest because it would enhance people’s resilience when facing stress and adversity (e.g., Neff 2003a; Gilbert 2005). This has been nicely demonstrated in a series of experimental studies conducted by Leary et al. (2007) who assessed the emotional and cognitive reactions of undergraduate students after being exposed to various types of unpleasant self-relevant events (e.g., receiving another person’s ambivalent feedback to one’s disclosure of a personal event). One of the main findings of these studies was that students scoring high on self-compassion exhibited less negative emotions and a more accepting attitude towards their failures than students scoring low on self-compassion.

The presumed resilience-promoting qualities of self-compassion also appear to have positive repercussions for people’s mental health. A recent meta-analytic study by MacBeth and Gumley (2012) identified 20 samples from 14 studies exploring the relation between self-compassion—as measured by Neff’s (2003b) Self-Compassion Scale—and common expressions of psychopathology, such as depression, anxiety, and stress. The results indicated that there was a robust and significant relationship between self-compassion and psychopathology in general (*r* = −.54), which was also evident for symptoms of depression (*r* = −.52), anxiety (*r* = −.51), and stress (*r* = −.54). As expected, the direction of this relationship was negative, which means that higher levels of self-compassion are generally accompanied by lower levels of psychopathological symptoms. Altogether, MacBeth and Gumley (2012; p. 550) conclude that these results provide support for “the importance of self-compassion for developing well-being, reducing anxiety and depression, and increasing resilience to stress.”

Adolescence is a challenging developmental period during which youths typically experience stress in relation to academic performance, interactions with parents, the position within the peer group, and their body image and sexual attractiveness (Santrock 2013). A substantial proportion of the adolescents suffers from emotional problems such as anxiety and depression (e.g., Clark et al. 1994; Lewinsohn et al. 1993), and so—given its association with resilience—self-compassion might be a relevant construct to explore within the context of adolescent mental health. So far, however, only a handful of studies have examined the relationship between self-compassion and psychological problems in adolescent populations. In the first study, Neff and McGehee (2010) administered the Self-Compassion Scale (SCS; Neff 2003b), the Beck Depression Inventory (Beck and Steer 1987), and the Trait form of the State-Trait Anxiety Inventory (Spielberger et al. 1970) in a sample of 235 high school students aged 14–17 years. Results indicated that there were substantial negative correlations between self-compassion on the one hand, and trait anxiety (*r* = −.73) and depressive symptoms (*r* = −.60) on the other hand, and these correlations were highly comparable to those documented in a comparison group of 287 young adults aged 19–24 years who were also included in this study (*r*’s being −.67 and −.51, respectively). In further research by Bluth and Blanton (2014a), an online survey including the SCS, the negative affect scale of the Positive and Negative Affect Scale (Watson et al. 1988), and an index of perceived stress was completed by a sample of 67 high school students aged 14–18 years. Robust negative correlations were found between self-compassion and negative affect (which includes a mixture of aversive mood states) and perceived stress (*r*’s being −.64 and −.70, respectively; see for similar findings: Bluth and Blanton 2014b). A study by Marshall et al. (2015) conducted in a large sample of 2448 Australian adolescents (mean age of 14–15 years) found a positive relationship between self-compassion as measured by the 12-item short form of the SCS (Raes et al. 2011) and mental health (*r* = .39). A follow-up assessment, 1 year later, demonstrated that self-compassion was also predictive of greater improvement in mental health, especially in vulnerable adolescents who were characterized by low levels of general self-esteem. In another investigation of 16- to 18-year-old male adolescents (*N* = 251) who dropped out of school and attended a voluntary residential program, Barry et al. (2015) also found that self-compassion was negatively related to various mental health indices, including vulnerable narcissism, aggression, anxiety, and depression. A final relevant study was performed by Zeller et al. (2015) who followed a group of 64 15- to 19-year-old adolescents who had been exposed to a traumatic event (i.e., a forest fire). Results indicated that self-compassion had protective properties with respect to resilience to and recovery from traumatic stress. More specifically, high levels of self-compassion were prospectively associated with lower levels of post-traumatic stress symptoms, panic complaints, depression, and suicidality. Thus, the few studies that have been conducted in young people demonstrate that self-compassion in adolescents relates in a similar way to emotional problems as in adults: that is, higher levels of self-compassion are associated with lower levels of anxiety, depression, and other psychological problems, and vice versa.

The present investigation further examined the relationship between self-compassion and emotional problems in adolescents. One-hundred-and-thirty-two high school students aged between 12 and 17 years from varying educational levels completed a modified version of the SCS (Neff 2003b) to assess individual differences in self-compassion as well as questionnaires for measuring symptoms of anxiety and depression. The SCS was adjusted in two ways: (1) items of the original SCS contain rather abstract formulations (e.g., “be understanding and patient to those aspects of my personality I don’t like”, “see my feelings as part of the human condition”, and “keep things in perspective”) that were considered as too abstract and rather obscure for the younger participants in our sample and especially those students with a lower educational level. Thus, it was decided to simplify the items of the SCS and if this was not possible to delete them from the questionnaire. (2) In keeping with Neff’s (2003b) initial conceptualization, the original SCS [and this was also true for the short form that was construed by Raes et al. (2011)] is not only composed of items referring to components that load positively on self-compassion (i.e., self-kindness, common humanity, and mindfulness), but also contains items pertaining to components that load negatively on this construct (i.e., self-judgment, isolation, and over-identification). It has been noted by various authors that these ‘negative’ components of self-compassion tend to inflate the relationship with anxiety and depression as they are more strongly related to such symptoms than the ‘positive’ components (e.g., Barnard and Curry 2011; see for examples Mills et al. 2007; Petrocchi et al. 2014; Ying 2009). Even more importantly, when regarding self-compassion as a protective mechanism within the context of mental health problems, it seems most appropriate to merely focus on the positive key features of self-compassion, and for this reason we discarded the negative items of the SCS. These modifications resulted in a brief nine-item questionnaire, the Shortened Self-Compassion Scale for Adolescents (S-SCS-A), with three items each for assessing the three positive components of self-kindness, common humanity, and mindfulness.

Using the S-SCS-A, we explored the relationship between self-compassion and adolescents’ anxiety and depression symptoms. We not only related the total self-compassion score to symptoms of anxiety and depression, but also investigated the relations among the three specific components of self-compassion and these common emotional symptoms. Such analysis may yield interesting information on which component(s) of self-compassion is (are) most relevant for psychopathology, but surprisingly so far the majority of studies did not look at the separate relations among self-kindness, common humanity, and mindfulness and mental health problems (for exceptions, see Mills et al. 2007; Petrocchi et al. 2014; Ying 2009) and this is also true for most investigations involving adolescents (but see Bluth and Blanton 2014b). An additional, interesting element of the current study was that we also included scales for measuring two other self-related constructs. The first one was self-esteem, a self-related concept for which a number of definitions have been put forward in the psychological literature (Swann et al. 2007). We focused on global self-esteem, which refers to a person’s overall cognitive and emotional evaluation of his or her own worth across various domains (Harter 1999). Although global self-esteem is considered to be less stable and more reactive than self-compassion (Neff and Vonk 2009), both concepts are viewed as highly relevant for a person’s general feeling of self-worth and as such it is not surprising that their link has often been studied in previous research (e.g., Barry et al. 2015; Marshall et al. 2015; Neff 2003b; Neff et al. 2007; Neff and Vonk 2009). The second construct was self-efficacy, which has to do with the individual’s perception of his or her ability to produce a desired action (Bandura 1997). Global self-esteem and self-efficacy have been shown to be important correlates and even predictors of mental health, and this is also true in youths where higher levels of these self-related constructs are typically accompanied by lower levels of anxiety and depression (e.g., Bandura et al. 1999; Cole et al. 1999; Evans et al. 1994; Muris 2002). Further, it has been demonstrated that both global self-esteem (e.g., Neff 2003b; Neff and Vonk 2009) and self-efficacy (Smeets et al. 2014) are positively related to self-compassion, indicating that these self-related concepts share similar features. Thus, in order to study the unique relation between self-compassion and mental health problems, it seems important to control for global self-esteem and self-efficacy. In previous studies with adults, some researchers have already demonstrated that the link between self-compassion and symptoms of anxiety and depression remains significant when controlling for global self-esteem (e.g., Neff 2003b; Neff et al. 2007), yet no investigation can be found that performed such an analysis while partialling out the influence of self-efficacy. We anticipated that even when controlling for global self-esteem and self-efficacy, there would still be a significant link between self-compassion and these mental health problems.

## Method

### Participants and Procedure

One-hundred-and-thirty-two adolescent participants (56 boys and 76 girls) aged between 12 and 17 years (*M* = 14.8 years, *SD* = 1.09) were recruited from three different high schools in the Southern part of the Netherlands (i.e., ‘Emma College’ in Hoensbroek, ‘Trevianum’ in Sittard, and ‘Het College’ in Weert). Most participants were from original Dutch descent (i.e., >90 %), and all of them had a good mastery of the Dutch language. Participants from three educational levels were included: 16.7 % followed low- or middle-level preparatory vocational education, 34.1 % higher general continued education, and 49.2 % pre-university secondary education. No exact information on the socio-economic status of the participants was available, but based on the occupations of both parents, it was estimated that 20.5 % of the participants had a low, 58.3 % a middle, and 21.2 % a high socio-economic background. The study was approved by the Ethical Committee of Psychology (ECP) at Maastricht University.

The directors of the schools were approached by telephone and after they decided to cooperate, 550 students were approached by sending their parents an information letter that also contained a consent form. About one quarter of the parents (i.e., 24 %) signed the form granting their child to participate in the study. These participants completed the set of questionnaires and a sheet with background information (such as age, sex, educational level, and the professions of their parents) during regular classes at school. The completion of the full survey lasted for about 20 min. All participants received a small reward (i.e., snack) in return for their participation. In addition, after the final testing session there was a lottery during which 10 participants could win a film voucher (€10).

### Questionnaires

#### Self-Compassion

As already noted in the introduction, self-compassion was assessed with a shortened and modified version of the SCS, the S-SCS-A. The procedure of modification was guided by the input of three young adolescents aged 12–15 years from various educational levels who were asked to read the items carefully with the goal of identifying the ‘difficult’ items of this adult questionnaire. A panel of three psychologists then simplified and modified the pertinent items. The nine items that were finally included in the S-SCS-A can be found in the “Appendix”. For each item, the young person has to respond on a five-point scale with 1 = *never* and 5 = *always*. A total self-compassion score and scores for the three subscales (i.e., self-kindness, common humanity, and mindfulness) can be computed by summing across relevant items. A psychologist who was familiar with the concept of self-compassion and the SCS had no difficulties in linking the S-SCS-A items to the three positive components of the original scale, which at least provides some support for the face validity of the modified version. Preliminary evidence for the reliability and validity of the S-SCS-A was found in a separate sample of 12- to 16-year-old adolescents (*N* = 184; Muris and Meesters, unpublished data). In that study, Cronbach’s alpha coefficients of .84 for the total scale and between .70 and .81 for the three subscales were found. Further, the correlation between the S-SCS-A and the 12-item short form SCS for adults (SF-SCS; Raes et al. 2011) appeared to be .62 (*p* < .001), and as expected this link was more carried by the positive subscales of the SF-SCS (*r* = .71) than by its negative subscales (*r* = .30; *Z* = 5.75, *p* < .001). Finally, as expected, adolescents had more difficulties to complete the adult SF-SCS, as evidenced by a higher frequency of missing values and questions asked about (difficult) items during the assessment session.

#### Self-Esteem

Global self-esteem was measured by means of a subscale of the Self-Perception Profile for Children (SPPC; Harter 1985). This subscale contains six items that each consist of two opposite descriptions, e.g., “Some kids are often unhappy with themselves” but “Other kids are pretty pleased with themselves”. Participants first choose the description that best fits and then indicate whether the description is *somewhat true* or *very true* for them. Accordingly, each item is scored on a four-point scale. A total score can be computed, with a higher score reflecting a more positive view of oneself. Psychometric evaluation of the SPPC has indicated that this scale provides a reliable and valid index of global self-esteem in children and adolescents (e.g., Harter 1985; Muris et al. 2003).

#### Self-Efficacy

The Self-Efficacy Questionnaire for Children (Muris 2001) is composed of 24 items assessing perceptions of self-efficacy in three domains: (1) social self-efficacy (eight items) which has to do with the perceived capability for dealing in an effective way with other people (e.g., “How well can you become friends with other children?”); (2) academic self-efficacy (eight items) which is concerned with the perceived capability to manage one’s academic affairs (e.g., “How well can you study when there are other interesting things to do?”); and (3) emotional self-efficacy (eight items) which pertains to the perceived capability of coping with negative emotions (e.g., “How well can you control your feelings?”). Each item has to be scored on a five-point scale with 1 = *not at all* and 5 = *very well*. A total self-efficacy score can be computed by summing all items. Research has yielded support for the reliability and validity of the SEQ-C (Muris 2001; Suldo and Shaffer 2007).

#### Anxiety

The latest revision of the Screen for Child Anxiety Related Emotional Disorders (SCARED; Birmaher et al. 1997) is a 75-item self-report questionnaire for measuring childhood anxiety disorders symptoms in terms of the Diagnostic and Statistical Manual of Mental Disorders, fifth edition (DSM-5; American Psychiatric Association 2013). Item examples are “I am afraid that something bad happens so I’ll never see my parents again”, “I worry about everything”, and “I am afraid I’ll do something embarrassing”. Children have to indicate how frequently they experience each symptom on a four-point scale: 0 = never, 1 = sometimes, 2 = often, and 3 = always. SCARED total and subscale scores can be obtained by summing relevant items. Previous research has demonstrated that the SCARED has good internal consistency, test–retest reliability, and validity (Birmaher et al. 1997; Muris et al. 2002).

#### Depression

The *Children’s Depression Inventory* (CDI; Kovacs 1981) is a commonly used self-report measure of depressive symptoms in children and adolescents 7–17 years of age. The scale has 27 items dealing with sadness, self-blame, loss of appetite, insomnia, interpersonal relationships, and school adjustment. Sample items are “I am sad all the time” and “I feel like crying every day”. CDI items are scored on three-point scales (0 = *not true*, 1 = *somewhat true*, 2 = *very true*). A total CDI score can be calculated by summing all item scores and varies between 0 (no depression symptoms) and 54 (all depression symptoms clearly present). The psychometric properties of the CDI have been tested extensively and are found to be adequate in clinical and non-clinical samples of children and adolescents (e.g., Saylor et al. 1984).

## Results

### Factor Structure and Reliability of the S-SCS-A

A principal components analysis (with Oblimin rotation) of the S-SCS-A yielded three factors with eigenvalues exceeding 1.00 (i.e., 3.98, 1.40, and 1.05). Together, these factors accounted for 71.44 % of the variance. Inspection of this three factor structure indicated that 8 out of 9 items loaded most substantially on their intended factor: items 2, 5, and 8 constituted the first factor of common humanity (factor loadings being .84, .88, and .70, respectively), items 1, 4 and 7 clearly loaded on the second factor of self-kindness (factor loadings being .92, .89, and .62, while items 3 and 9 convincingly loaded on the third factor of mindfulness (factor loadings being .84 and .92, respectively). Item 6 (i.e., “When things go wrong, I try to say to myself that it might still be worse”) was the only exception to this rule: this item did significantly load (i.e., .46) on its hypothesized factor of mindfulness, but had a more substantial loading (i.e., .68) on the common humanity factor. Nevertheless, based on the positive face validity check by our expert, we decided to include this item in the mindfulness subscale.

The internal consistency reliability of the S-SCS-A was satisfactory. Cronbach’s alphas were .84 for the total scale and all >.70 for the subscales (see Table 1), which can be qualified as good certainly when one keeps in mind that the subscales only consist of a limited set of (three) items.

**Table 1:** Mean scores (standard deviations) and reliability coefficients for the Shortened Self-Compassion Scale for Adolescents (S-SCS-A) and the other questionnaires used in the present study

	Total sample (*N* = 132)	Boys (*n* = 56)	Girls (*n* = 76)	Cronbach’s α
S-SCS-A self-compassion	28.73 (6.54)	29.14 (6.65)_a_	28.43 (6.48)_a_	.84
S-SCS-A self-kindness	9.27 (2.91)	9.54 (2.92)_a_	9.08 (2.91)_a_	.79
S-SCS-A common humanity	9.26 (2.74)	9.25 (2.72)_a_	9.26 (2.77)_a_	.78
S-SCS-A mindfulness	10.20 (2.51)	10.36 (2.48)_a_	10.09 (2.55)_a_	.74
SPPC self-esteem	18.32 (4.17)	19.93 (4.00)_a_	17.13 (3.90)_b_	.94
SEQ-C self-efficacy	87.02 (16.45)	92.09 (14.82)_a_	83.29 (16.69)_b_	.94
SCARED anxiety	32.78 (25.45)	20.75 (20.44)_a_	41.64 (25.25)_b_	.96
CDI depression	6.84 (7.87)	4.88 (7.58)_a_	8.29 (7.82)_b_	.93

*SPPC* self-perception profile for children, *SEQ*-*C* self-efficacy scale for children, *SCARED* screen for child anxiety related emotional disorders, *CDI* children’s depression inventory

Means not sharing similar subscripts indicate a significant gender difference at *p* < .05

### Other General Findings

Other questionnaires that were used in this study also displayed good reliability, with all Cronbach’s alphas being >.90 (see Table 1). Further, significant gender differences were found for global self-esteem [*t*(130) = 4.03, *p* < .001], self-efficacy [*t*(130) = 3.14, *p* < .01], anxiety disorders symptoms [*t*(128.80, adjusted *df*) = 5.01, *p* < .001], and depressive symptoms [*t*(130) = 2.51, *p* < .05]. As can be seen in Table 1, boys had higher levels of global self-esteem and self-efficacy than girls, whereas girls exhibited higher symptom levels of anxiety disorders and depression compared to boys. Note also that in general no gender differences were documented for self-compassion [all *t*(130)’s < 1]. However, when analyzing the data of younger (13- and 14-year-old) and older (15- to 17-year-old) adolescents separately, a significant gender difference in self-compassion did emerge in older youth. That is, among 15- to 17-year-olds, boys displayed significantly higher levels of total self-compassion [means being 31.54, *SD* = 6.11 vs. 27.97, *SD* = 6.84, *t*(64) = 2.19, *p* < .05] and self-kindness in particular [means being 10.29, *SD* = 2.80 vs. 8.89, SD = 2.76, *t*(64) = 2.01, *p* < .05] than did girls. Finally, no significant relations between age and self-compassion scores were found in this sample (all *r*’s ≤ .08).

### Relations Among Self-Compassion, Other Self-Related Constructs, and Symptoms

Correlations (controlled for gender) between self-compassion as measured by the S-SCS-A and scales assessing other self-related constructs and symptoms of anxiety disorders and depression are shown in Table 2. Four conclusions can be drawn from this table. First, inter-correlations among S-SCS-A subscales ranged between .37 and .55 (all *p*’s < .001), which indicates that various components of self-compassion were positively related to each other. Second, self-compassion was positively related to other self-related constructs: the total score of the S-SCS-A correlated .44 with global self-esteem and .50 with self-efficacy (both *p*’s < .001). As for S-SCS-A subscales it was found that mindfulness showed the strongest links with global self-esteem and self-efficacy (*r*’s being .54 and .63, respectively, both *p*’s < .001), while common humanity was least clearly correlated with these self-related constructs (*r*’s being .17, non-significant, and .28, *p* < .01, respectively). Third, significant negative correlations were found between self-compassion on the one hand and scales for measuring anxiety disorders and depressive symptoms on the other hand. More precisely, the total S-SCS-A score correlated −.26 (*p* < .01) with anxiety and −.35 (*p* < .001) with depressive symptoms. Correlations between S-SCS-A subscales and symptom measures revealed that it was mainly the mindfulness component that carried the significant relations between self-compassion and symptoms of anxiety and depression (*r*’s being −.34 and −.45, both *p*’s < .001). The other self-compassion components were less convincingly linked to symptom levels: nonetheless, self-kindness was significantly negatively associated with depressive symptoms (*r* = −.28, *p* < .01), while common humanity had a small but significant negative relation to anxiety symptoms (*r* = −.19, *p* < .05). The relative importance of the mindfulness component was also confirmed by means of regression analyses in which symptoms scores were predicted from the three S-SCS-A subscales (and gender). That is, in the case of both anxiety and depression, mindfulness was the only self-compassion component explaining a significant proportion of symptom scores (β’s being −.34 and −.50, respectively, both *p*’s < .001). Fourth and finally, significant negative correlations were also found among global self-esteem and self-efficacy on the one hand and symptoms of anxiety disorders and depression on the other hand (all *r*’s between −.45 and −.67, all *p*’s < .001).

**Table 2:** Correlations (corrected for gender) among the Shortened Self-Compassion Scale for Adolescents (S-SCS-A) and other questionnaires

	(1)	(2)	(3)	(4)	(5)	(6)	(7)
S-SCS-A self-compassion							
S-SCS-A self-kindness	.78***						
S-SCS-A common humanity	.80***	.37***					
S-SCS-A mindfulness	.83***	.47***	.55***				
SPPC self-esteem	.44***	.37***	.17	.54***			
SEQ-C self-efficacy	.50***	.32***	.28**	.63***	.75***		
SCARED anxiety	−.26**	−.12	−.19*	−.34***	−.45***	−.58***	
CDI depression	−.35***	−.28**	−.12	−.45***	−.67***	−.65***	.66***

*SPPC* self-perception profile for children, *SEQ*-*C* self-efficacy scale for children, *SCARED* screen for child anxiety related emotional disorders, *CDI* children’s depression inventory

* *p* < .05; ** *p* < .01; *** *p* < .001

### Unique Links Between Self-Compassion and Symptoms While Controlling for Other Self-Related Constructs

Given the overlap among the three self-related constructs as well as the finding that each of them was negatively associated with symptom levels, regression analyses were carried out to explore the unique links between self-compassion, global self-esteem, and self-efficacy on the one hand and anxiety and depressive symptoms on the other hand. As we were most interested in the contribution of self-compassion to symptom levels beyond other self-related constructs, a hierarchical approach was chosen in which self-compassion was added to the model (on step 3) after entering gender (step 1) and self-efficacy and global self-esteem (step 2). As shown in Table 3, the first analysis predicting anxiety disorders symptoms revealed that on step 1 gender made a significant contribution to the model (β = .41, *p* < .001), accounting for 17 % of the variance in anxiety scores. On step 2, only self-efficacy explained a unique and significant proportion of the variance (β = −.53, *p* < .001), explaining an additional 28 % of the variance. Most importantly, entering self-compassion on step 3 did not make a significant contribution, showing that this self-related construct did not account for variance in anxiety scores beyond the other variables in the model.

**Table 3:** Results of the regression analyses predicting symptoms of anxiety disorders and depression from self-compassion and other self-related constructs

	B	SE	β	Δ*R* ^2^
SCARED anxiety				
Gender	20.90	4.11	.41**	.17**
SEQ-C self-efficacy	−.83	.16	−.53**	.28**
SPPC self-esteem	−.14	.64	−.03	
S-SCS-A self-compassion	.13	.30	.03	.00
CDI depression				
Gender	3.41	1.36	.22*	.05*
SEQ-C self-efficacy	−.16	.05	−.34**	.48**
SPPC self-esteem	−.82	.18	−.44**	
S-SCS-A self-compassion	.00	.09	.00	.00

*S*-*SCS*-*A* Shortened Self-Compassion Scale for Adolescents, *SPPC* self-perception profile for children, *SEQ*-*C* self-efficacy scale for children, *SCARED* screen for child anxiety related emotional disorders, *CDI* children’s depression inventory

* *p* < .05; ** *p* < .001

The regression analysis predicting depressive symptoms yielded a similar result. On step 1 gender made a significant contribution (β = .22, *p* < .05), whereas on step 2 both self-efficacy (β = −.34, *p* < .001) and global self-esteem (β = −.44, *p* < .001) were found to explain a significant and unique proportion of the variance in CDI scores. Both steps accounted for respectively 5 and 48 % of the variance in depression scores. However, as can be seen in the bottom panel of Table 3, on step 3 self-compassion again did not have a unique impact on depression symptoms once controlling for gender and the other self-related constructs.

Similar regression analyses predicting anxiety and depression symptom levels from S-SCS-A subscales revealed comparable findings. These analyses indicated that none of the self-compassion components was capable of explaining a significant and unique proportion of the variance in anxiety and depressive symptoms when controlling for gender and the other self-related constructs.

Finally, as self-efficacy emerged as a significant (unique) correlate of both anxiety disorders and depressive symptoms and because this construct also consists of three components (i.e., social, academic, and emotional self-efficacy), additional regression analyses were carried out including the SEQ-C subscales as predictor variables while controlling for gender, self-compassion and global self-esteem. The regression model predicting anxiety symptoms revealed that only emotional self-efficacy made a unique significant contribution (β = −.67, *p* < .001), whereas in the model predicting depression academic and emotional self-efficacy emerged as independent significant predictors (β’s being −.18, *p* < .05, and −.34, *p* < .01, respectively) .

## Discussion

This study further examined the relations between self-compassion and symptoms of anxiety disorders and depression in a sample of non-clinical youths. On first sight, the present findings are nicely in keeping with what has been documented in previous research conducted in adult (see the review by MacBeth and Gumley 2012) and adolescent (Barry et al. 2015; Bluth and Blanton 2014a, b; Marshall et al. 2015; Neff and McGehee 2010; Zeller et al. 2015) populations. That is, negative correlations were found between self-compassion and scales for measuring anxiety and depression, indicating that higher levels of self-compassion were associated with lower symptom levels, and vice versa.

It should be noted that the magnitude of the correlations between self-compassion and anxiety and depression in this adolescent sample was considerably smaller than that reported in the meta-analysis [by MacBeth and Gumley (2012); *r*’s being −.26 and −.35 vs. −.51 and −.52, respectively]. A first possible explanation is developmental in nature and has to do with the idea that self-compassion may not have been fully crystallized in this sample of young adolescents. That is, it has been argued that (early) during the developmental stage of adolescence, youths are still very egocentric (e.g., Elkind 1967), implying that they become easily absorbed by their own difficulties, take negative things very personally, and have problems to reflect objectively on and distance themselves from such issues, thereby hindering the mechanism of self-compassion to occur. As a related point, research has shown that anxiety and depressive symptoms peak around age 15 or 16 (Cohen et al. 1993), and so it may well be that emotional symptoms levels were still rather low in this population, which contained quite a number of younger adolescents. Altogether, the relatively young adolescents in this sample may have had fairly low levels of both self-compassion and emotional symptoms, resulting in an attenuation of the mutual correlations among these constructs. A second explanation has to do with the method of this study and more precisely with the instrument that we employed to measure self-compassion. Given the fairly young age and the low educational level of some participants, we decided to shorten and modify Neff’s (2003b) SCS. The resulting S-SCS-A contained only nine items referring to components that load positively on self-compassion (i.e., self-kindness, common humanity, and mindfulness)—thereby doing more justice to the ‘protective’ nature of the concept, while discarding items pertaining to components having a negative load on this construct (i.e., self-judgment, isolation, and over-identification, which are included in the original SCS as well as in its short form). Several authors (Barnard and Curry 2011; Petrocchi et al. 2014) have noted that when only using the positive indicators of self-compassion, relationships with mental health symptoms remain present but tend to be less robust. Thus, while being aware of the fact that the original construct of self-compassion includes positive as well as negative indicators, one wonders whether—within the context of mental health problems—it seems preferable to employ a scale only measuring the protective elements (i.e., positive indicators) of the construct.

Some evidence for the psychometric qualities of the S-SCS-A was obtained in this study. First of all, a principal components analysis performed on the items of this scale revealed three factors that corresponded rather well with the three hypothesized components of self-compassion, namely self-kindness, common humanity, and mindfulness. Second, the S-SCS-A proved to be reliable in terms of internal consistency. A Cronbach’s alpha of .84 was found for the total scale, while this reliability coefficient ranged between .74 and .79 for the three subscales. Third and finally, evidence was also found for the validity of the S-SCS-A. That is, substantial positive correlations were found between the S-SCS-A and the other self-related constructs of global self-esteem and self-efficacy. There was only one exception to this rule. That is, the common humanity subscale did not correlate significantly with global self-esteem, probably because self-esteem is more concerned with comparing oneself to others rather than feeling connected to others (which is reflected in this specific component of self-compassion). Nevertheless, in general, the correlations between self-compassion and other self-related constructs were in keeping with what has been reported in the adult literature (Neff 2003b; Neff and Vonk 2009; Smeets et al. 2014) and the magnitude of the inter-correlations underlines that self-compassion, self-esteem, and self-efficacy are distinct but related aspects of the personal identity (Leary and Tangney 2012).

Within a context of mental health problems, most studies conducted so far have focused on the total construct of self-compassion, thereby neglecting the contributions of the separate components of self-kindness, common humanity, and mindfulness (for exceptions, see Mills et al. 2007; Petrocchi et al. 2014; Ying 2009) and this also pertains to the investigations that have been conducted in adolescent populations (but see Bluth and Blanton 2014b). The current findings indicate that it was in particular the mindfulness component of the self-compassion construct that was most convincingly associated with symptoms of anxiety disorders and depression. This suggests that this component, which has to do with keeping a balanced view when experiencing adversity and not getting totally absorbed by the problems (Neff 2003a, b), seems more relevant than the other components of self-kindness and common humanity in this adolescent sample. In passing, it is important to note that the mindfulness component of self-compassion only reflects a rather specific aspect of the much broader concept of mindfulness that has been described elsewhere in the psychological literature. For example, Baer et al. (2006) identified five facets of mindfulness (i.e., describing, observing, acting with awareness, non-judging, and non-reactive) that are all concerned with the ability of “bringing one’s complete attention to the experiences occurring in the present moment, in a non-judgmental or accepting way” (p. 27).

Psychopathological phenomena such as (adolescent) anxiety disorders and depression usually have a multifactorial origin not only involving vulnerability but also protective variables (e.g., Wenar and Kerig 2000), and among the latter category self-related constructs are thought to play an important role (Baumeister and Vohs 2004). Many of these constructs have been studied in isolation, and so it is important to carry out research in which multiple concepts are examined in relation to psychological symptoms in order to learn more about their relative and unique influences. So far, in relation to symptoms of anxiety and depression, self-compassion has been compared against global self-esteem (Neff 2003b; Neff et al. 2007) and general mindfulness (Bluth and Blanton 2014a; Van Dam et al. 2011), and this research has generally revealed that the construct indeed has incremental predictive value. However, the present findings indicate that when controlling for other self-related constructs, and in particular self-efficacy, self-compassion no longer showed a unique link to symptoms of anxiety and depression. Again, the aforementioned developmental and methodological explanations can be put forward to account for this result, but it is good to keep in mind that previous research has also demonstrated that self-efficacy—and especially emotional self-efficacy—is a particularly strong correlate of anxiety and depression, even when controlling for other etiological factors (Muris et al. 2011; Rudy et al. 2012; Suldo and Shaffer 2007). This fits nicely with Bandura’s (1997) notion that “the belief about the ability to perform behaviors that bring desired outcomes” is an important determinant of personal functioning. Although research in the past has primarily focused on relevance of self-efficacy in an academic context (Multon et al. 1991), studies increasingly explore emotion-related self-efficacy and its relation to psychological well-being in youth (see Valois et al. 2015). In the meantime, it is also important to keep in mind that with these cross-sectional data it is not possible to convincingly test temporal relations among self-compassion, other self-related constructs, and emotional symptoms. Although our findings suggest that self-compassion does not explain variance in symptoms *beyond* other self-related constructs, the construct might still be relevant because its relation to symptoms of anxiety and depression may be exerted via mediation by other self-related variables. Obviously, future prospective studies are needed to explore this possibility.

Two additional findings deserve some further comment. To begin with, in keeping with what has been reported elsewhere in the literature, significant gender differences were found for global self-esteem, self-efficacy, and anxiety and depressive symptoms, with girls displaying lower levels of self-esteem (cf. Muris et al. 2003) and self-efficacy (cf. Muris 2002), but higher levels of anxiety and depressive symptoms (cf. Muris 2002) as compared to boys. When analyzing the total sample of adolescents, no gender differences were documented for self-compassion, which is at odds with what has been found in adult samples—where women typically display somewhat lower levels of self-compassion than men (e.g., Neff 2003b). However, when analyzing the data of younger and older adolescents separately, it was found that—among older youth—girls indeed exhibited lower levels of self-compassion than boys. A similar finding was documented by Bluth and Blanton (2014b) and is in keeping with the notion of a gradual crystallization of self-compassion during the developmental stage of adolescence. Further, the results of the regression analyses indicated that whereas anxiety disorders symptoms were only uniquely predicted by self-efficacy, depressive symptoms were predicted by self-efficacy as well as global self-esteem. This seems to suggest that both types of emotional problems are characterized by deficits in the perceived ability of producing desirable outcomes, but that only depressive symptoms are accompanied by diminished global self-worth, which seems to be in accordance with the diagnostic criteria as formulated in the DSM (APA 2013). Finally, it is also puzzling why emotional—and to a lesser extent—academic self-efficacy were uniquely linked to symptoms of anxiety and depression while social self-efficacy was not. Being accepted by the social environment (i.e., peers) is an important developmental challenge for young people (Santrock 2013), and so one wonders why social self-efficacy did not play a role. Additional correlations revealed that emotional and social self-efficacy were substantially correlated (*r* = .70), and thus it is likely that they both competed for the same variance in symptom scores. It is not that surprising that in an analysis explaining youths’ emotional symptoms, the feeling that one is capable of regulating one’s emotions (i.e., emotional self-efficacy) turned out to be more relevant than the feeling that one is capable of engaging adequately in social interactions (i.e., social self-efficacy). If we had investigated the contributions of these self-efficacy components to—let’s say—quantity and quality of friendships, social self-efficacy would probably have been a better predictor than emotional self-efficacy.

It should be acknowledged that the present study suffers from various limitations. First and foremost, as noted earlier we employed a cross-sectional design, which means that no conclusions can be drawn on the cause-effect relation between self-compassion, other self-related constructs, and symptoms of anxiety and depression. Second, only about a quarter of the participants who were approached actually participated in this research by completing the set of questionnaires, which of course raises doubts on the generalizability of the current findings to the entire adolescent population. Third, this research was conducted in a European country (i.e., The Netherlands), while most previous research on self-compassion in youth has been conducted in the United States. It is possible that inconsistencies between findings of this study and earlier work reflect cultural differences. Fourth and finally, this investigation also relied on a non-clinical sample, and so it would be interesting to conduct this study in youths displaying clinical levels of anxiety and depression. Self-compassion, self-esteem, and self-efficacy all seem to be good for the self, and so it is worthwhile to know exactly what the relationships are among these constructs and which of these self-related constructs matter the most in youths actually suffering from these common types of psychopathology as this may also provide important leads for treatment.

## Appendix

Items of the Shortened Self-Compassion Scale for Adolescents (S-SCS-A) and corresponding items (and numbers) from the original scale.

1. When I feel sad, I try to be tender to myself.
  I try to be loving towards myself when I am feeling emotional pain (SCS-5).
2. When I have problems, I remind myself that everybody has difficulties from time to time.
  When things are going badly for me, I see the difficulties as part of life that everyone goes through (SCS-3).
3. When something upsets me, I am also able to think about things that are still going well.
  When something upsets me, I try to keep my emotions in balance (SCS-9).
4. When I feel unhappy, I try to be kind to myself.
  I’m kind to myself when I’m experiencing suffering (SCS-19).
5. When I feel sad, I remind myself that I am not the only person on the world feeling this way.
  When I am down and out, I remind myself that there are lots of other people in the world feeling like I am (SCS-7).
6. When things go wrong, I try to say to myself that it might still be worse.
  When something painful happens, I try to take a balanced view of the situation (SCS-14).
7. When I am going through a hard time, I take good care of myself.
  When I am going through a hard time, I give myself the caring and tenderness I need (SCS-12).
8. When I handle things the wrong way, I remind myself that everybody makes mistakes from time to time.
  When I feel inadequate in some way, I remind myself that feelings of inadequacy are shared by most people (SCS-10).
9. When I am feeling down, I am still able to think about positive things.
  When I am feeling down, I try to approach my feelings with curiosity and openness (SCS-22).

*Note* Items 1, 4 and 7 = self-kindness; items 2, 5, and 8 = common humanity; items 3, 6, and 9 = mindfulness. *SCS* Self-Compassion Scale (Neff 2003b).
